# High-fat diet feeding and palmitic acid increase CRC growth in β2AR-dependent manner

**DOI:** 10.1038/s41419-019-1958-6

**Published:** 2019-09-26

**Authors:** Sarwat Fatima, Xianjing Hu, Chunhua Huang, Weixiong Zhang, Jing Cai, Min Huang, Rui-Hong Gong, Minting Chen, Alan H. M. Ho, Tao Su, Hoi Leong Xavier Wong, Zhaoxiang Bian, Hiu Yee Kwan

**Affiliations:** 1 Centre for Cancer and Inflammation Research, School of Chinese Medicine, Hong Kong Baptist University, Hong Kong, China; 2 Institute of Chinese Medical Sciences, University of Macau, Macau, China; 3 Research Center for Ecology and Environmental Sciences, Northwestern Polytechnical University, Xi’an, China; 4 International Institute for Translational Chinese Medicine, Guangzhou University of Chinese Medicine, Guangzhou, Guangdong 510006 China

**Keywords:** Hormones, Colon cancer

## Abstract

Epidemiology studies indicate that consumption of high-fat diet (HFD) is directly associated with the development of colorectal cancer (CRC). However, the exact component in HFD and the mechanism underlying its effect on CRC growth remained unclear. Our study shows that HFD feeding increases β2AR expression in the xenograft tissues of CRC-bearing mouse model; the elevated β2AR expression is reduced when HFD is replaced by control diet, which strongly suggests an association between HFD feeding and β2AR expression in CRC. HFD feeding increases palmitic acid and stearic acid levels in CRC; however, only palmitic acid increases β2AR expression, which is dependent upon Sp1. β2AR plays the dominant role in promoting CRC cell proliferation among all the β-AR subtypes. More importantly, knockout of β2AR or knockdown of Sp1 abolishes the palmitic acid increased CRC cell proliferation, suggesting palmitic acid increases CRC cell proliferation in β2AR-dependent manner. HFD or palmitic acid-rich diet (PAD) also fails to increase the tumor growth in xenograft mouse models bearing β2AR-knockout CRC cells. β2AR promotes CRC growth by increasing the phosphorylation of HSL at the residue S552. The phosphorylated and activated HSL (S552) changes the metabolic phenotype of CRC and increases energy production, which promotes CRC growth. Our study has revealed the unique tumorigenic properties of palmitic acid in promoting CRC growth, and have delineated the underlying mechanism of action. We are also the first to report the linkage between HFD feeding and β-adrenergic signaling pathway in relation to CRC growth.

## Background

Colorectal cancer (CRC) is one of the most common cancers in the world and presents one of the highest rates of morbidity and mortality worldwide^1^. There were over 1.8 million new cases in 2018. Overall, the lifetime risk of developing CRC is around 1 in 22 (4.49%) for men and 1 in 24 (4.15%) for women.

The incidence of CRC is strongly influenced by nutrition and high-fat or high-carbohydrate western-style diet^2^. Epidemiology studies also indicate that consumption of the western-style diet is directly associated with the development of CRC^3^; in which high-fat diet (HFD) contributes to 80% of the CRC cases^4^. Although the connection between HFD consumption and CRC development is known, the interpretation of the effect of each of the dietary component in HFD on the cancer growth is limited. For example, experimental studies only show that consumption of HFD will favor lipid oxidation which yields the products 4-hydroxynonenal and oxysterols that are the risk factors for inflammation and CRC development^5^. HFD also induces LGR5 expression and hence induces colon carcinogenesis^6^. Another study shows that the saturated fat in HFD induces bile secretion into the intestine, which selects the gut microbes population and alters the bile acid pool. These changes lead to the production of tumor-promoting secondary bile acids such as deoxycholic acid and lithocholic acid^7^. While other studies are focused on whether and how the heme iron in the red meat in the HFD leads to the development of CRC^8^.

HFD is rich in saturated fatty acid such as palmitic acid. Emerging evidence showing palmitic acid serves not only a fatty acid to produce energy, but also an intracellular signaling molecule involved in the development of cancers^9^. Unlike other fatty acids, palmitic acid boosts the metastatic potential of melanoma and breast cancer cells in a CD36-dependent manner^10^; and also promotes the growth of prostate cancer by activating the signal transducer and activator of transcription 3^11^. However, the specific role of palmitic acid in promoting CRC growth is not known. Identification of the underlying mechanism of action will greatly enhance our understanding on how the component of HFD affects CRC growth.

Besides HFD consumption, CRC development is also influenced by physiological stress. Same as other cancers, CRC patients receive substantial stress during diagnosis and treatment, and this psychological stress is associated with the cancer progression and mortality^12,13^. The physiological stress elevates the catecholamine levels in cancer patients^14^. These catecholamine bind onto the β-adrenergic receptors (β-AR) and activate the β-adrenergic signaling pathway, and hence affect cancer pathogenesis^13^. However, up to present, factors other than psychological stress that will activate β-adrenergic signaling pathway in CRC has never been explored. In this study, we are the first to reveal the linkage between HFD consumption and β-adrenergic signaling pathway in CRC, in which HFD feeding or palmitic acid increases CRC growth in a β2AR-dependent manner.

## Results

### HFD feeding increases β2AR expression in CRC

**Fig. 1:**
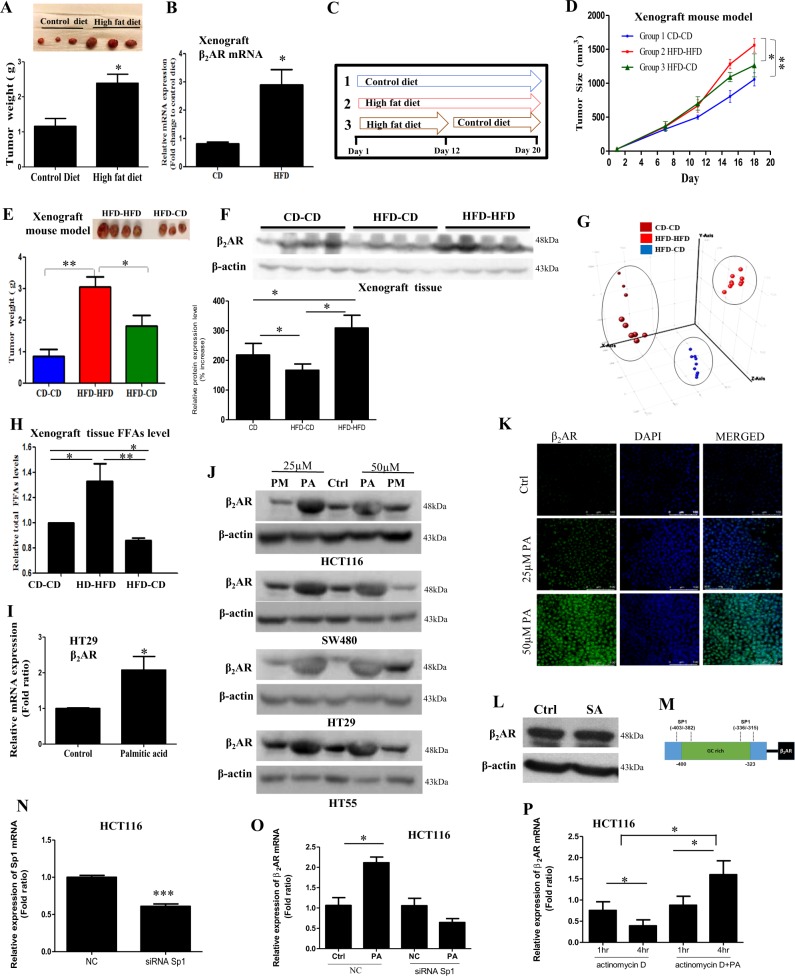
High-fat diet (HFD) feeding or palmitic acid treatment increases β2AR expression in CRC. **a** Tumor weight and **b** mRNA expression of β2AR in the xenograft tissues of the control diet (CD)-feedind and HFD-feeding CRC-bearing xenograft mouse models. **c** The dietary intervention for the CRC-bearing xenograft mouse models. **d** Tumor size and **e** tumor weight of the CRC-bearing xenograft mouse models under the dietary intervention. **f** The β2AR protein expression in the xenograft tissues of the CRC-bearing xenograft mouse model under the dietary intervention, and quantification of the expression levels. **g** Clustering of the lipid samples of the xenograft tissues revealed by principle component analysis and **h** free fatty acid (FFA) levels in the xenograft tissues of the CRC-bearing mouse model under the dietary intervention. **i** β2AR mRNA expression and **j** protein expression in CRC cells treated with (PA) palmitic acid, or (PM) treated with PA followed by replacing the culture medium devoid of PA. **k** Immunofluorescence staining showing β2AR expression in CRC cells treated with PA. **l** β2AR protein expression in CRC cells treated with stearic acid (SA, 25 µM). **m** Schematic illustration of the GC-rich enhancer region (−400/−323) of β_2_AR promoter region showing two evolutionarily conserved Sp1 binding sites (−403/−382 and −336/−315). **n** mRNA level of Sp1 in CRC cells after transfected with negative control (NC)-siRNA or Sp1-siRNA. **o** mRNA level of β2AR in CRC cells treated with PA (25 µM) after NC-siRNA or Sp1-siRNA transfection **p** mRNA level of β2AR in CRC cells treated with actinomycin D (3ug/ml) in the presence or absence of PA (25 µM). CD–CD mice kept feeding control diet, HFD–CD high-fat diet changed to control diet, HFD–HFD mice kept feeding high-fat diet. Data are represented as mean ± SEM, ^*^p < 0.05. ^**^p < 0.01, *n* = 3 individual experiments, or 3–9 mice in each group

To explore the mechanism of action underlying how HFD promotes CRC growth, we first established a HFD-associated CRC xenograft mouse model. We subcutaneously inoculated HCT116 CRC cells into the male nude mice. When the tumors were grown to about 50 mm^3^ in size, we randomly divided these mice into two groups, the HFD-feeding group and the matched control diet (CD)-feeding group. We found that the tumor weights were significantly higher in the HFD-group than those in the CD group after 12 days of the dietary intervention (Fig. 1a).

We then performed a high-throughput screening of the oncogenic signaling molecules with these xenograft tissues. We found that β2-adrenergic receptor (β2AR) mRNA expression in the xenograft tissues of the HFD-group were significantly higher than those in the CD group (Fig. 1b). To further suggest whether the expression of β2AR is correlated with HFD feeding, we modified the experimental design. On day 12, we randomly selected some of the mice from the HFD-group and replaced the HFD by CD (Fig. 1c, lane 3, HFD–CD); while the other mice kept feeding HFD (Fig. 1c, lane 2). Interestingly, the tumor size (Fig. 1d), tumor weight (Fig. 1e), and tumor β2AR protein expressions (Fig. 1f) were reduced after HFD was replaced by CD. These data strongly suggest an association between HFD feeding and β2AR expression in CRC.

We next asked how HFD feeding increased β2AR expression in CRC. Since the HFD and CD have different fatty acid profiles (Supplementary Table 1), we explored whether the fatty acid contents in the xenograft tissues were also different under different dietary intervention, and whether these fatty acid(s) affected β2AR expression in CRC. Our lipidomics data showed that the xenograft tissue lipid profiles were affected by the diets as revealed by the sample clustering in principle component analysis (Fig. 1g). Furthermore, total fatty acid levels in the xenograft tissues were significantly higher in HFD-group when compared to CD group (Fig. 1h); and the elevated fatty acid levels were reduced after HFD was replaced by CD (HFD–CD) (Fig. 1h). To identify which fatty acid species in tumor tissues were affected by diets, we used Agilent Mass Profiler Professional software (Agilent Technologies) coupled with LIPID MAPS database to identify the fatty acid species. We also performed targeted lipidomics to quantify and validate the changes of these fatty acid candidates. We found that palmitic acid and stearic acid levels were elevated in the xenograft tissues upon HFD feeding; the elevated levels were significantly reduced after HFD was replaced by CD (Table 1). Although the level of oleic acid was also high in HFD, its level in the tumor tissues was not elevated under HFD feeding (Table 1).

**Table 1:** Quantification of palmitic acid, stearic acid, and oleic acid in xenograft tissues

ng/mg protein xenograft tissues	Pamitic acid	Stearic acid	Oleic acid
CD-CD	1.0204 ± 0.0280	0.5120 ± 0.0181	4.0387 ± 0.0201
HFD-HFD	4.6550 ± 0.0232^a^	14.0742 ± 0.3258^a^	0.1180 ± 0.0055^a^
HFD-CD	3.0331 ± 0.1777^b,d^	1.2206 ± 0.0292^b,c^	0.0967 ± 0.0045^a^
Retention time (min)	17.541	19.615	17.898
Accurate MS	256.2402	284.2715	282.468

*n* = 5–9 mice in each group. a < 0.01 compared to CD–CD group; b < 0.05 compared to CD–CD group, c < 0.01 compared to HFD–HFD group, d < 0.05 compared to HFD–HFD group. CD–CD mice kept feeding control diet, HFD–CD high-fat diet changed back to control diet, HFD–HFD mice kept feeding high-fat diet

To examine whether the saturated fatty acids palmitic acid and stearic acid affected β2AR expression in CRC, we used CRC cell models for the study. Palmitic acid significantly increased β2AR mRNA expression (Fig. 1i) but not β1AR mRNA expression (Supplementary Fig. S1). We could not detect β3AR mRNA expression in the CRC cell lines (data not shown). Palmitic acid also significantly increased β2AR protein expression in CRC cells (Fig. J labeled PA and Fig. K). To further suggest the increased β2AR expression was associated with palmitic acid treatment, we first treated the CRC cells with palmitic acid for 24 h, and then cultured these cells in culture medium devoid of palmitic acid (Fig. 1j, labeled PM) for another 24 h before we examined the β2AR expression. We found that removal of palmitic acid from the culture medium significantly reduced the β2AR protein expression in these cells (Fig. 1j and Supplementary Fig. S2). These data suggest an association between β2AR expression and palmitic acid treatment. Interestingly, stearic acid did not affect β2AR expression in CRC cells (Fig. 1l).

Next, we examined how palmitic acid increased β2AR expression in CRC. The human β2AR is encoded by the gene ADRB2 on chromosome 5q31–32. Study shows that GC-rich element in the promoter region of ADRB2 harbors the conserved binding sites for Sp1 which upregulate ADRB2 gene transcription^15^ (Fig. 1m). Our data showed that palmitic acid did not affect Sp1 expression in CRC cells (Supplementary Fig. S3). However, in siRNA-mediated Sp1 knockdown CRC cells (Fig. 1n), palmitic acid failed to increase β2AR mRNA expression (Fig. 1o), suggesting palmitic acid increases β2AR expression in a Sp1-dependent manner.

We also explored other possibility that may change the β2AR mRNA level. We examined whether palmitic acid affected ADRB2 mRNA half-life. We blocked the cellular transcription with inhibitor actinomycin D, which is known to interfere with transcription by intercalating into DNA. We found that palmitic acid significantly increased β2AR expression in the CRC cells under actinomycin treatment (Fig. 1p), suggesting palmitic acid increases β2-AR mRNA half-life.

### Among the β-AR subtypes, β2AR plays a dominant role to promote CRC growth

**Fig. 2:**
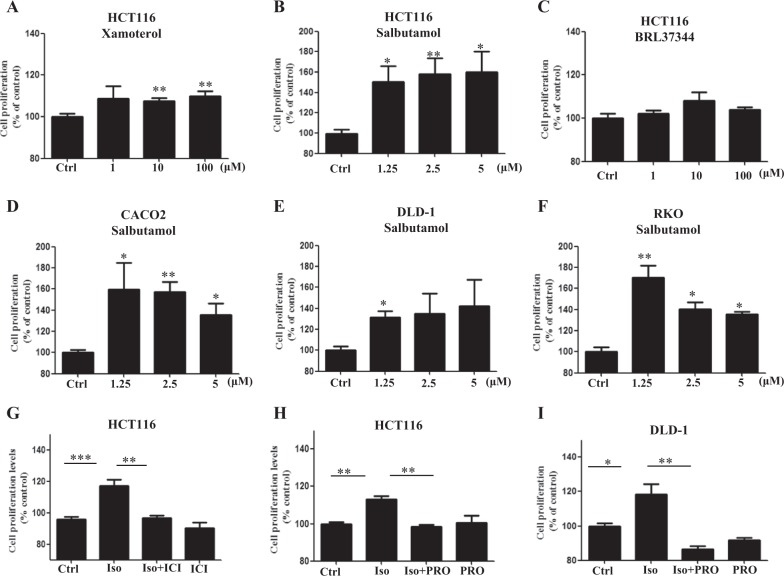
Among the β-AR subtypes, β2AR plays a dominant role to promote CRC growth. Proliferation of HCT116 cells treated with **a** xamoterol, **b** salbutamol (SAL), and **c** BRL37344 at the indicated concentrations for 24 h. **d**–**f**, Proliferation of CRC cells treated with SAL at the indicated concentrations for 24 h. **g** Proliferation of HCT116 cells treated with or without isoprenaline (Iso) (10 µM) in the presence or absence of ICI-118,551 (5 µM), for 24 h. **h**, **i** Proliferation of HCT116 and DLD-1 cells treated with or without Iso (10 µM) in the presence or absence of propranolol (PRO) (80 µM), for 24 h. **J** Data are represented as mean ± SEM, ^*^p < 0.05. ^**^p < 0.01, *n* = 3 individual experiments

Since both HFD feeding and palmitic acid increased the β2AR expression, we investigated the role of β2AR in CRC cell proliferation in comparison to other β-AR subtypes. We separately treated the CRC cells with subtype β-AR agonizts which included xamoterol for β1AR, salbutamol for β2AR and BRL37344 for β3AR. Data showed that activation of β2AR by salbutamol resulted in the greatest increase in cell proliferation compared to xamoterol and BRL37344 (Fig. 2a–c). The increased proliferation upon β2AR activation was also demonstrated in other CRC cell lines (Fig. 2d–f). To further suggest the dominant role of β2AR in promoting CRC cell proliferation, we treated the cells with β-AR agonist isoprenaline (Iso) in the presence or absence of specific β2AR antagonist ICI-118,551. We found that ICI-118,550 completely abolished the Iso-increased CRC cell proliferation (Fig. 2g). Similarly, another β2AR antagonist propranolol also significantly reduced the Iso-increased CRC cell proliferation (Fig. 2h, i, I). Both isoprenaline and propranolol did not significantly affect the basal cell proliferation (Fig. 2g–i). Taken together, these data suggest the important role of β2AR in promoting CRC cell proliferation.

### HFD feeding and palmitic acid increase CRC growth in a β2AR-dependent manner

**Fig. 3:**
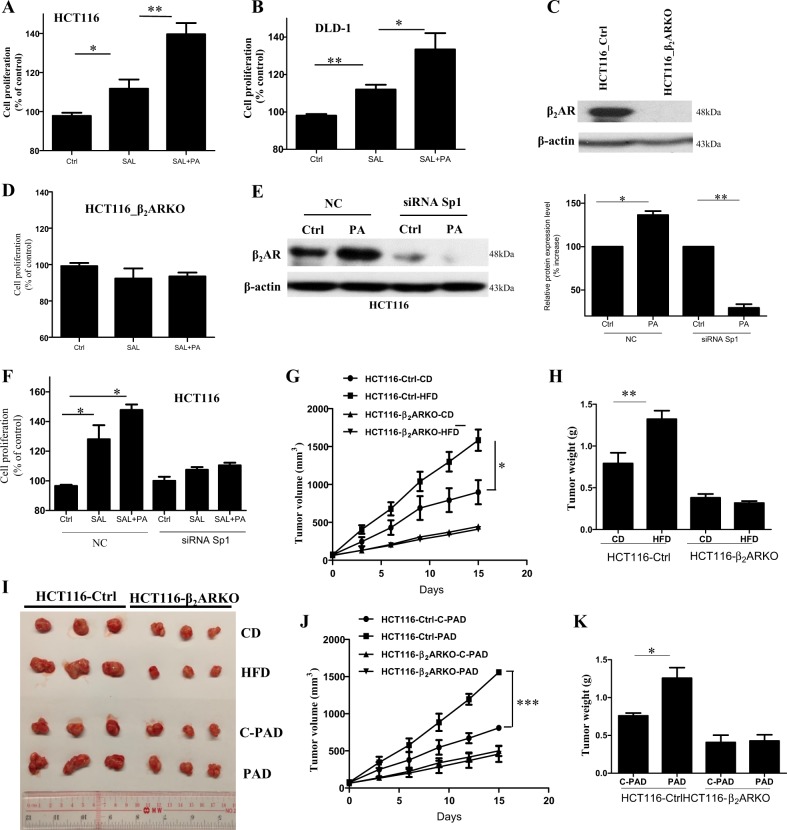
HFD feeding and palmitic acid increase CRC growth in β2AR-dependent manner. Proliferation of **a** HCT116 and **b** DLD-1 cells treated with salbutamol (SAL) (1.25 µM) in the presence or absence of PA (25 µM), for 24 h. **c** CRISPR/Cas9 system-mediated knockout of β_2_AR in HCT116 cells (HCT116-β_2_ARKO). **d** Proliferation of HCT116-β_2_ARKO cells treated with SAL (1.25 µM) in the presence or absence of PA (25 µM), for 24 h. **e** Expression of β_2_AR protein in HCT116 cells after negative control (NC)-siRNA or Sp1-siRNA transfection, with or without PA treatment, and quantification of the expression levels. **f** Proliferation of HCT116 cells transfected with NC-siRNA or Sp1-siRNA, after treated by SAL (1.25 µM) in the presence or absence of PA (25 µM), for 24 h. **g** Volume and **h** weight of the tumors in the xenograft mouse models bearing either control HCT116 cells or HCT116-β_2_ARKO cells, fed by either control diet (CD) or high-fat diet (HFD). **j** Volume and **k** weight of the tumors in the xenograft mouse models bearing either control HCT116 cells or HCT116-β_2_ARKO cells, fed by either palmitic acid-rich diet (PAD) or its corresponding control diet (C-PAD). **i** The tumor of these xenograft mouse models. Data are represented as mean ± SEM, ^*^p < 0.05. ^**^p < 0.01, ^***^p < 0.001, *n* = 3 individual experiments, or 3–9 mice in each group

Our data suggest that palmitic acid increases β2AR expression which is important in promoting CRC cell proliferation. If this is true, palmitic acid should further increase β2AR-mediated CRC cell proliferation. Indeed, as shown in Fig. 3a, b, palmitic acid significantly enhanced salbutamol-mediated CRC cell proliferation. To further suggest the role of β2AR in palmitic acid-enhanced CRC cell proliferation, we performed a CRISPR/Cas9 system-mediated knockout of β2AR in CRC cells (HCT116_ β2ARKO) (Fig. 3c). We found that in HCT116_ β2ARKO cells, salbutamol and palmitic acid failed to increase CRC cell proliferation (Fig. 3d). Since our previous data showed that palmitic acid increased β2AR expression in a Sp1-dependent manner, we also used siRNA to knockdown Sp1 in CRC cells. As shown in Fig. 3e, knockdown of Sp1 significantly reduced both the basal and palmitic acid-enhanced β2AR expression in CRC cells. In these Sp1-knockdown cells, salbutamol and palmitic acid failed to increase CRC cell proliferation (Fig. 3f). These in vitro data clearly demonstrated that palmitic acid increased CRC cell proliferation in a β2AR-dependent manner.

To suggest the role of β2AR in HFD-enhanced CRC growth in vivo, we established two different xenograft mouse models. In model 1, mice were inoculated with control HCT116 cells. In model 2, mice were inoculated with HCT116_ β2ARKO cells. When the tumors of these mice grew to about 50 mm^3^ in size, we randomly divided the mice into HFD group and matched CD group. We found that HFD feeding could only increase the tumor size and weight of the xenograft mouse model which were inoculated with control HCT116 cells (model 1) (Fig. 3g–i). In mice inoculated with HCT116_ β2ARKO cells (model 2), HFD failed to increase the tumor size and weight (Fig. 3g–i). However, since HFD has other saturated fatty acids, we repeated the animal study with a palmitic acid-rich diet (PAD) (D16042106, Research Diets) and its corresponding CD (C-PAD) (D17042705, Research Diets) (Supplementary Table 1). Similarly, we found that PAD could only increase the tumor size and weight of the xenograft mouse models which were inoculated with control HCT116 cells (model 1) (Fig. 3i, j, k). In the xenograft models inoculated with HCT116_ β2ARKO cells (model 2), PAD failed to increase the tumor size and weight (Fig. 3I, j, k). Taken together, our data clearly showed that both HFD feeding and PAD-feeding increased CRC growth in a β2AR-dependent manner in vivo.

### Activation of β2AR activates the cAMP/PKA axis and increases hormone sensitive lipase (HSL) phosphorylation at S552 in CRC

**Fig. 4:**
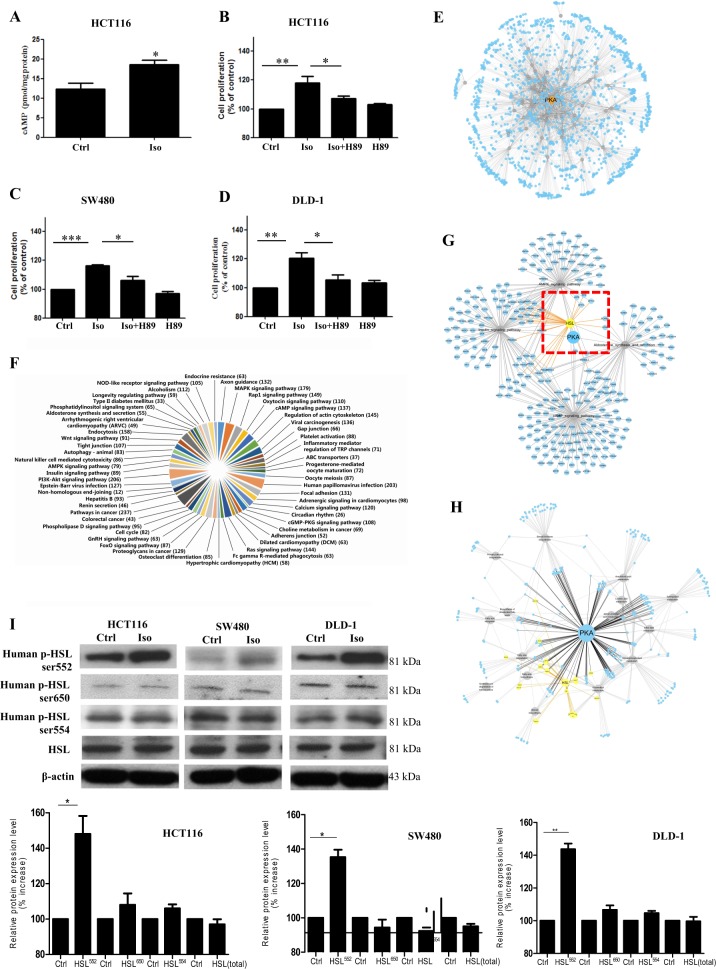
Activation of β2AR activates the cAMP/PKA axis and increases hormone sensitive lipase (HSL) phosphorylation at S552 in CRC. **a** cAMP levels in HCT116 cells after isoprenaline (Iso) treatment (10 µM) for 24 h. **b**–**d** Proliferation of CRC cells treated with or without Iso (10 µM) in the presence or absence of H89 (10 µM), for 24 h. **e** Bioinformatic screening using functional enrichment analysis for putative PKA targets and **f** various functions of the putative PKA targets. **g** Identification of HSL as one of PKA targets from several enriched pathways and **h** interaction network of HSL involved in human lipid metabolism. **i** Expression of total HSL and phosphorylated HSL (ser552, ser650, and ser554) in CRC cells treated with Iso (10 µM) for 24 h, and quantification of the expression levels. Data are represented as mean ± SEM, ^*^p < 0.05, ^**^p < 0.01, ^***^p < 0.001. *n* = 3 individual experiments

Since HFD feeding increases CRC growth in a β2AR-dependent manner, our next question is how β2AR activity affects CRC cell growth. β2AR belongs to the guanine nucleotide-binding G-protein-coupled receptor family; it is well-known that the cAMP/PKA axis is activated upon the receptor activation. Indeed, in CRC cells, we also showed that βAR activation increased cAMP level (Fig. 4a). To examine whether the cAMP/PKA axis played a role in CRC cell proliferation, we inhibited PKA activity in these cells with inhibitor. We found that β-adrenergic-enhanced CRC cell proliferation was reduced when PKA was inhibited (Fig. 4b–d), suggesting PKA activity is involved in the β-adrenergic-stimulated CRC cell proliferation.

Next, we used bioinformatics and system biology to identify putative PKA targets and investigated whether the highlighted target candidate(s) was crucial in the CRC cell proliferation. We performed bioinformatics screening of putative PKA targets with human protein data sets obtained from three authoritative databases, namely Ensemble, Swiss-Prot, and NCBI. PKA is a serine/threonine kinase. In the screening, PKA targets were defined as the protein sequences containing one or more PKA phosphorylation consensus sequences. The PKA phosphorylation consensus sequence, R/K-R/K-X-S/T^16^ was used for searching the human protein collections. We then performed functional enrichment analysis with these targets, the result was then visualized by Cytoscape software^17^ (Fig. 4e). These putative PKA targets involved in different physiological and pathological functions (Fig. 4f). Interestingly, the screening revealed more than one-third of these putative PKA targets are associated with lipid metabolism. Among all these target candidates, hormone sensitive lipase (HSL) was highlighted in four significantly enriched pathways, namely cAMP signaling pathway, insulin signaling pathway, AMPK signaling pathway and aldosterone synthesis and secretion pathway (Fig. 4g). In these enriched pathways, HSL activity is associated with lipid metabolism. The interaction network of HSL with the proteins related to lipid metabolism was also suggested (Fig. 4h).

We then found out which putative PKA phosphorylation site(s) on HSL was phosphorylated upon β-adrenergic stimulation in CRC cells. Interestingly, we found that only the phosphorylation at Ser552 was increased (Fig. 4i) upon β-adrenergic stimulation; other putative PKA phosphorylation sites (Ser650) or AMPK phosphorylation sites on HSL were not affected by the treatment (Fig. 4i).

### HSL phosphorylation at S552 changes the metabolic phenotype of the CRC cells and increases CRC cell proliferation

**Fig. 5:**
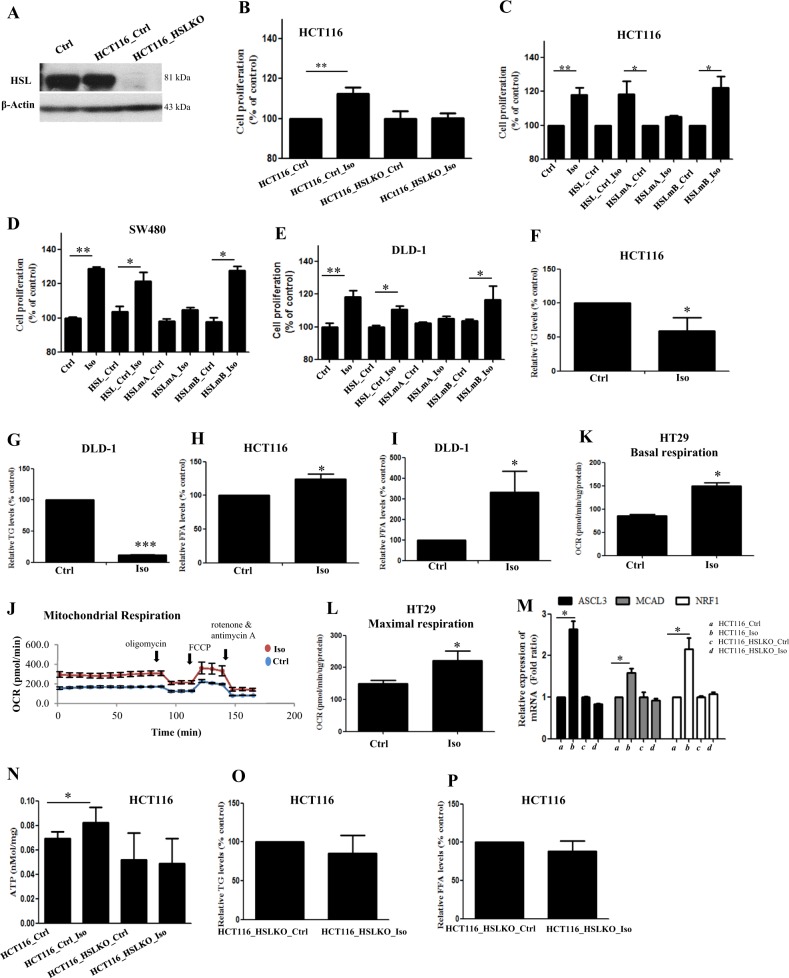
HSL phosphorylation at S552 changes the metabolic phenotype of the CRC cells and increases CRC cell proliferation. **a** CRISPR/Cas9 system-mediated knockout of HSL in HCT116 cells (HCT116_HSLKO). **b** Proliferation of control HCT116 cells and HCT116_HSLKO after Iso treatment (10 µM) for 24 h. **c**–**e** Proliferation of control CRC cells or CRC cells overexpressed with control empty vector (HSL-Ctrl) or HSL-S552A (HSLmA) or HSL-S650A (HSLmB) after Iso (10 µM) treatment for 24 h. **f**, **g** Triglyceride (TG) and **h**, **i** free fatty acid (FFA) levels in CRC cells after Iso treatment (10 µM) for 24 h. **j** Representative graph showing the oxygen consumption rate (OCR), and the **k** basal respiration and **l** maximal respiration of the CRC cells treated with Iso (10 µM), analyzed by Seahorse XFe24 analyzer. **m** The mRNA expressions of fatty acid oxidation genes, acyl-CoA synthetase long-chain family member 3 (ASCL3), medium-chain acyl-coenzyme A dehydrogenase (MCAD), and NF-E2-related factor 1 (NRF1) in control HCT116 cells and HCT116_HSLKO cells after Iso treatment (10 µM) for 24 h. **n** Intracellular ATP levels of the control HCT116 cells or HCT116_HSLKO cells after Iso treatment (10 µM) for 24 h. **o** Triglyceride (TG) and **p** free fatty acid (FFA) levels in HCT116_HSLKO cells after Iso treatment (10 µM) for 24 h

We next asked whether HSL activity is required and whether HSL phosphorylation at S552 is critical for CRC growth. In this regard, we used CRISPR/Cas9 system to mediate HSL knockout in CRC cells (HCT116_HSLKO) (Fig. 5a). We found that β-adrenergic stimulation failed to increase HCT116_HSLKO cell proliferation (Fig. 5b). Besides, inhibition of HSL activity by Cay10499 also reduced the β-adrenergic-increased CRC cell proliferation; while Cay10499 treatment alone did not affect basal CRC cell proliferation (Supplementary Fig. S4). We also performed site-directed mutagenesis to mutate S522 on HSL (S552A). Our data clearly showed that S552A, but not the mutation of S650 residue (S650A), completely abolished the β-adrenergic-stimulated CRC cell proliferation (Fig. 5c–e). Taken together, these data suggest that HSL phosphorylation at S552 is critical to mediate the CRC cell proliferation.

However, how HSL activity affects CRC cell proliferation? HSL is a lipolytic enzyme which mobilizes FFA from triglyceride (TG), the released FFA will be used as fuel to produce energy for cancer growth^9^. Indeed, we also found that activation of HSL by β-adrenergic stimulation reduced TG levels (Fig. 5f, g), increased cellular FFA level (Fig. 5h, i), changed the metabolic phenotypes of the CRC cells (Fig. 5j), increased both basal (Fig. 5k) and maximal respiration (Fig. 5l), increased β-oxidation gene expressions (Fig. 5m) and ATP production (Fig. 5n). While in HCT116_HSLKO cells, β-adrenergic stimulation failed to reduce TG level (Fig. 5o), and also failed to increase FFA level (Fig. 5p), β-oxidation gene expressions (Fig. 5m), and ATP production (Fig. 5n).

## Discussion

**Fig. 6:**
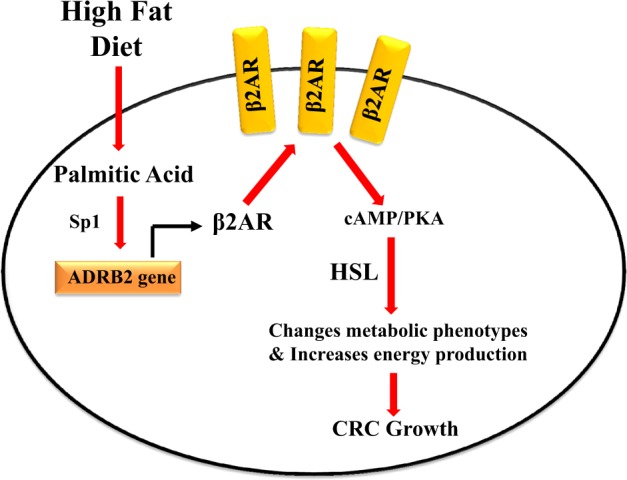
HFD feeding and palmitic acid increase CRC growth in β2AR-dependent manner. A diagram illustrating how HFD increases CRC growth. HFD feeding increases palmitic acid level in CRC. Palmitic acid increases the β2AR expression in CRC cells in a Sp-1 dependent manner. Upon activation of the β2AR, cAMP/PKA axis is activated, which increases the phosphorylation of HSL at S552. The phosphorylated HSL (S552) mediates changes of the metabolic phenotypes and increases energy production in CRC, which promotes CRC growth

Our data clearly show that HFD feeding increases palmitic acid level in CRC. And palmitic acid increases β2AR expressions in CRC cells in Sp-1 dependent manner. Upon activation of β2AR by β-adrenergic agonist, which are always elevated in cancer patients^13^, cAMP/PKA axis is activated, and hence increases the phosphorylation of HSL at S552. The phosphorylated HSL (S552) mediates changes of the metabolic phenotypes and increases energy production in CRC, which promotes CRC growth (Fig. 6). Our study has revealed a novel mechanistic pathway delineating how palmitic acid, the dominant saturated fatty acid in HFD, promotes CRC growth.

We are the first to report the linkage between HFD feeding and β-adrenergic signaling pathway in relation to CRC growth. Our data have also dissected which HFD component per se, promotes CRC growth. Palmitic acid is the very common saturated fatty acid found in many dietary fats such as lard, butter, peanut oil, tallow, soybean, and also olive oil^9^. Palmitic acid in cancer cells can come from endogenous synthesis and exogenous uptake. Our study shows that upon HFD feeding, palmitic acid and stearic acid levels in CRC are significantly increased, suggesting CRC can uptake these exogenous fatty acids. However, stearic acid fails to increase β2AR expressions and CRC cell proliferation (data not shown). Our data suggest the uniqueness of palmitic acid in its tumorigenic properties. It has been reported that palmitic acid specifically boosts the metastatic potential of CD36+ metastasis-initiating cells in a CD36-dependent manner^10^ and increases the metastatic potential of many cancers such as melanoma and breast cancer^10^. Other fatty acids such as oleic and linoleic acid^18^ only contribute to the cellular lipid pool and increase fatty acid oxidation^19^.

Our data show that palmitic acid increases β2AR expression which is dependent on Sp1, knockdown of Sp1 abolishes the palmitic acid increased β2AR expression. β2AR is the dominant β-AR subtype in promoting CRC growth although controversy exists among different studies. Some experimental studies show that suppression of β2AR but not β1AR signaling selectively suppresses CRC cell viability and inhibits CRC growth; however, others report that both β1 and β2 antagonists inhibit CRC growth^14,20,21^. No matter whether β1AR activity is involved or not, β2AR activation promotes CRC growth. β2AR is activated by catecholamine which levels in CRC patients are always elevated^14^ due to the substantial psychological stress. However, whether palmitic acid or other component in HFD promotes the synthesis or secretion of catecholamines in CRC patients remains unknown, which deserves further investigation.

In our study, we show that palmitic acid increases β2AR expression in CRC. However, we have not examined whether palmitic acid per se activates β2AR or increases the responsiveness of β2AR to β2-agonizts. β2AR will be desensitized and internalized after agonist-induced stimulation. Study shows that PKA and G protein coupled receptor kinase GRK2 and GRK6 desensitize β2AR upon its activation by β2-agonist^22,23^. PKA phosphorylation induces β2AR switch in coupling from Gαs to Gαi protein, which terminates the cAMP-mediated downstream signaling pathway. GRKs phosphorylation of β2AR creates binding site for β-arrestin which induces receptor internalization^23^. No study has been done to examine whether palmitic acid per se or HFD feeding affects the activity of these protein kinases. Understanding whether and how palmitic acid affects β2AR internalization will have an important implication not only in the field of oncology, but also neurobiology.

Although epidemiological studies have confirmed the association between β-adrenergic stimulation and CRC growth, the underlying mechanism of action is less studied. Our data suggest HSL is the downstream mediator promotes CRC growth upon β2AR stimulation. Epidemiological studies have linked the usage of β-blockers to reduce the rate of progression of several solid tumors, and the β-antagonist seems to be more effective in inhibiting cancer at the early stage as revealed by preclinical laboratory models and human pharmaco-epidemiological studies. Preclinical pharmacological studies are now laying the groundwork for the translation of β-blockers as a novel adjuvant to the existing therapeutic strategies in clinical oncology^24^. However, it is well-known that β-AR antagonists have severe side-effects including bronchospasm, heart failure, prolonged hypoglycemia, bradycardia, heart block, intermittent claudication and Raynaud’s phenomenon; while neurological reactions, include depression, fatigue, and nightmares^25,26^. Our work may have suggested HSL inhibitors could be a viable alternative to β-AR antagonists as a safer therapeutics for CRC patients who are under psychological stress or have to intake diet with a relatively high-fat content due to other complications such as diabetes mellitus.

In conclusion, our study clearly shows that HFD feeding or palmitic acid increases CRC growth which is β2AR-dependent. Our study has paved the path for the exploration of the interrelated effects of HFD feeding and psychological stress on CRC growth.

## Materials and methods

### Materials

Antibodies for western blot, including HSL, p-HSL (phosphorylation at Ser563, Ser565, and Ser 660), and β-Actin were purchased from Cell Signaling Technology. Antibody against β2-adrenergic receptor was purchased from Abcam. Isoprenaline (Iso), Cay10499 (Cay), H89, propranolol (PRO), norepinephrine, salbutamol (SAL), BRL37344, palmitic acid (PA), and fatty acid free-bovine serum albumin (BSA) were purchased from Sigma–Aldrich. ICI-118,551 (ICI) was purchased from Caymen Chemical.

### Cell lines

CRC cell lines HCT116, SW620, SW480, and DLD-1 were purchased from American Type Culture Collection (ATCC). They were cultured in Dulbecco’s modified essential medium supplemented with 10% fetal calf serum (Life Technologies Limited), and 1% penicillin and streptomycin (Life Technologies Limited). The cell lines were maintained in a humidified incubator at 37 °C with 5% carbon dioxide.

### Cell proliferation

CRC cells were seeded in 96-well plates at a density of 3000 cells/well. The following day, cells were incubated with 10 µm isoprenaline (Iso) for 24 h. For HSL inhibitor and β-AR antagonist studies, cells were preincubated with 2 µm Cay10499 (Cay) or 10 µm H89 or 80 µm propranolol (PRO) for 12 h before Iso treatment. For β-AR agonist studies, cells were treated by xamoterol, salbutamol (SAL), and BRL37344, respectively, for 24 h at the indicated concentrations. For PA treatment, cells were treated with 50 µm PA in the presence of 1% fatty acid free-BSA for 48 h, 1% fatty acid free-BSA alone served as control. Proliferation was measured by Invitrogen^™^ CyQUANT^™^ Cell Proliferation Assay which stains the DNA in the cells. Since the amount of DNA in each cell remains constant, so assays based on DNA content can provide an accurate and simple measure of cell number, that indicate the cell proliferation. We performed the assay following the Company’s instruction.

### Bioinformatics searching of PKA putative targets

Bioinformatics searching of PKA putative targets was conducted by using the PKA consensus sequence, R/K-R/K-X-S/T in which X represented any amino acids. Human protein data sets were obtained from three well-known public databases, Ensemble, Swiss-Prot, and NCBI. Proteins that contained one or more PKA consensus sequences were considered as putative PKA targets. Then KEGG and GO enrichment analysis were carried out with the putative targets to predict functional categories involving PKA. Cytoscape software was used to visualize the putative targets and functional pathways related to PKA.

In silico screening of PKA targets was carried out again with proteins involving in 15 human lipid metabolism pathways found in KEGG database. Interactions of HSL with the proteins relating to human lipid metabolism were searched in String database. Both results were integrated and visualized by Cytoscape software.

### Western blotting analysis

The nitrocellulose membrane carrying transferred proteins was incubated at 4 °C overnight with corresponding antibody at 1:1000 ratio. Immunodetection was accomplished using horseradish peroxidase-conjugated secondary antibody, followed by ECL detection system (Amersham).

### siRNA transfection

Transient transfections of siRNA was done using Lipofectamine RNAiMAX (Invitrogen) transfecting reagent according to manufacturer’s instructions. Briefly, CRC cells were seeded in six-well plates and transfected using different siRNA concentrations and 5 μL Lipofectamine RNAiMAX for 24 h.

### Real-time polymerase chain reaction analysis

Total RNA was extracted with Trizol reagent (Invitrogen) and treated with DNAase 1 (Invitrogen). RNA (1 μg) was reverse transcribed with oligo-dT using M-MLV reverse transcriptase (Promega) according to manufacturer’s protocol. Real-time polymerase chain reaction (PCR) was performed using SYBR green reaction mixture in the ABI 7500 fast real-time PCR system (Applied Biosystems). The gene expression data was normalized to the endogenous control β-actin. The relative expression levels of genes were measured according to the formula 2-ΔCt, where ΔCt is the difference in threshold cycle values between the targets and β-actin. All samples were analyzed in triplicate. Primers used for this study are as follows: β2AR forward: 5-AATAGCAACGGCAGAACGGA-3, β2AR reverse: 5-TCAACGCTAAGGCTAGGCAC-3; Sp1 forward: 5-G G C T A C C C C T A C C T C A A A G G-3, Sp1 reverse: 5-C A C A A C A T A C T G C C C A C C A G-3;acyl-CoA synthetase long-chain family member 3 (ASCL3) forward: 5-T G T G C G A C A G C T T T G T T T T C-3, ASCL3 reverse: 5-C T G A C C A A C A G G A C A G C A G A-3; medium-chain acyl-coenzyme A dehydrogenase (MCAD) forward: 5-T T G A G T T C A C C G A A C A G C A G-3, MCAD reverse: 5-A G G G G G A C T G G A T A T T C A C C-3; NF-E2-related factor 1 (NRF1) forward: 5-A G C A A A A G C A G A G G G T T T C A-3, NRF1 reverse: 5-C T G T G T T T G C G T T T G C T G A T-3.

### ATP analysis

ATP Bioluminescence assay kit (Beyotime Technology, China) was used to measure intracellular ATP levels. Following treatment with isoprenaline (Iso) for 24 h, cells were lysed and ATP levels were measured according to the manufacturer’s instructions.

### Mitochondrial respiration

The oxygen consumption rate (OCR) was measured using the XFe24 Extracellular Flux Analyzer (Agilent Technologies). HT29 cells were seeded in a 96-well plate at 10,000 cells/well and incubated overnight at 37 °C, 5% CO_2_ humidified atmosphere. One our prior to loading the cell plate into the Analyzer, cells were washed twice with pre-warmed, serum-free XF Base Assay Media (Agilent Technologies), supplemented with 2 mM l-glutamine, 1 mM sodium pyruvate, and 10 mM glucose (pH 7.4) and incubated at 37 °C for 1 h. Isoprenaline (Iso) was added to cells to a final concentration of 10 μM. OCR was quantified following consecutive treatment of HT29 cells with four treatments: (1) assay medium alone, (2) 2 μM oligomycin, (3) 1 μM FCCP, and (4) 5 μM rotenone and antimycin A. For each assay, individual basal measurements were taken, followed by consecutive injection of treatments. Measurements were recorded after each injection, with each measurement consisting of 10 s mixing and 3 min measurement period.

### Establishment of β2AR and HSL knockout clones

β_2_AR CRISPR-Cas9 KO plasmid (Santa Cruz), HSL CRISPR-Cas9 KO plasmid (Santa Cruz), β_2_AR HDR plasmid (Santa Cruz), and HSL HDR plasmid (Santa Cruz) were used for β_2_AR and HSL knockout in HCT116 cells, respectively (HCT116-β_2_ARKO, HCT116_HSLKO). HCT116 cells were grown to 80–90% confluence and transfected with the CRISPR plasmids by using UltraCruz transfection reagent (Santa Cruz) at room temperature. HCT116 control cells were transfected with pX330-U6-Chimeric_BB-CBh-hSpCas9 (HCT116_Ctrl). Cells were treated with 1 µg/ml puromycin (Sigma) for 3–4 weeks. Western blot analysis was used to confirm β_2_ARKO and HSL knockout in HCT116_Ctrl, HCT116-β_2_ARKO, and in HCT116_HSLKO cells. For transient transfection of HSL mutant plasmids (HSLmA, with mutation at HSL S552A, and HSLmB with mutation at HSL S650A) was done by Lipofectamine 2000. pCDNA3.1 vector served a control for transient transfection (HSL_Ctrl).

### cAMP determination

The cells were treated with Iso for 24 h and then washed with phosphate-buffered saline (PBS) and lysed for cAMP measurement with immunoassay kit (BioVision) following manufacturer’s instructions. The protein quantities of the cells were measured by the Bradford method. Each treatment was performed in triplicate.

### Sample preparation for LC/MS-based lipidomics

CRC cells were treated with 10 µM isoprelanine (Iso) for 24 h. Lipids were extracted from these cells for the lipidomics study. To each sample, we added 0.3 ml 0.5 M KH_2_PO_4_, 1.5 ml chloroform and 0.5 ml methanol. After vortex for 2 min and centrifugation at 2000 × g, the lower phase was collected and evaporated under a nitrogen stream. The residue was reconstituted in 100 μl of isopropanol-acetonitrile (1:9, v/v) for liquid chromatography mass spectrometry (LC/MS) analysis.

### LC/MS-based lipidomics analysis and data processing

An Agilent 6540 UHD Accurate-Mass Q-TOF LC/MS mass spectrometer (Agilent Technologies, USA) was connected to an Agilent 1290 Infinity UHPLC via an ESI ion source for the analysis of total lipids. An Agilent 6450 Triple Quadrupole LC/MS system accompanied with MassHunter Workstation software (Version B.04.00 Qualitative Analysis, Agilent Technologies) was connected to an Agilent 1290 Infinity UHPLC for specific quantification of targeted bioactive lipids and lipid metabolites. Briefly, we set up a gradient mobile phase comprising solvent TL-A (40% ACN with 10 mM ammonium acetate) and solvent TL-B (acetonitrile: isopropanol, 1:9) with 10 mM ammonium acetate. The raw data were first processed by MassHunter Workstation software (Version B.04.00 Qualitative Analysis, Agilent Technologies). Ions were extracted by molecular features characterized by retention time (RT), intensity in apex of chromatographic peak and accurate mass. These results were then analyzed by Mass Profiler Professional (MPP) software (Version 2.2, Agilent Technologies). We also set up a filtration platform to further filter the initial entities before doing principle component analysis. Only entities with abundances above 3000 cps were selected. These entities were then passed a tolerance window of 0.15 min and 2 mDa chosen for alignment of RT and *m*/*z* values, respectively. We employed one-way ANOVA to do the statistical analysis. The *p* value of ANOVA was set to be 0.05 (corresponding with the significance level of 95%).

### Nude mice xenograft model

Male nude mice (6 weeks old) were obtained from the Laboratory Animal Services Centre, Chinese University of Hong Kong. The nude mice were maintained in ventilated cages in a specific animal handling room of Hong Kong Baptist University. All care and handling of animals were performed with the approval of the Government of The Hong Kong Special Administrative Region Department of Health. HCT116_Ctrl, HCT116-β_2_ARKO, HCT116_HSLKOcells (1 × 10^6^) cells were resuspended in 0.1 ml PBS and inoculated subcutaneously into the backs of nude mice and allowed to grow for 7 days. After that, mice were randomly assigned to groups (*n* = 9 mice for each group) fed with HFD (D12492) or its matched CD (D12450J); or palmitic acid-rich diet (PAD, D16042106) or its corresponding CD (C-PAD, D17042705). All the diets were purchased from Research Diets, Inc. The mice were treated by intragastric administration with isoprenaline (Iso) or salbutamol (SAL) or vehicle control. Body weight and tumor volume were measured every day. Tumor volumes were determined by a caliper and calculated according to the following formula: (width^2^ × length)/2. At the end of the experiment, mice were sacrificed, and tumor xenografts were collected and weighed. Tumor tissues were stored at −80 °C for subsequent experiments.

### Statistical analysis

All data are expressed as mean ± SEM. Statistical analysis was performed by the Student’s *t* test, using the statistical software Graphpad Prism 5.0. *p* Value < 0.05 was considered statistically significant. All measurements were conducted at least in triplicates.

## Supplementary information

Supplementary Figures and Table
