# Supplementary material for: High-fat diet feeding and palmitic acid increase CRC growth in β2AR-dependent manner
Source: Cell Death Dis. 2019 Sep 26;10(10):711. doi: 10.1038/s41419-019-1958-6 (PMC6763436; doi:10.1038/s41419-019-1958-6)
Supplement: Supplementary file 1 — Supplementary Figures and Table [file 41419_2019_1958_MOESM1_ESM.ppt]

## Slide 1
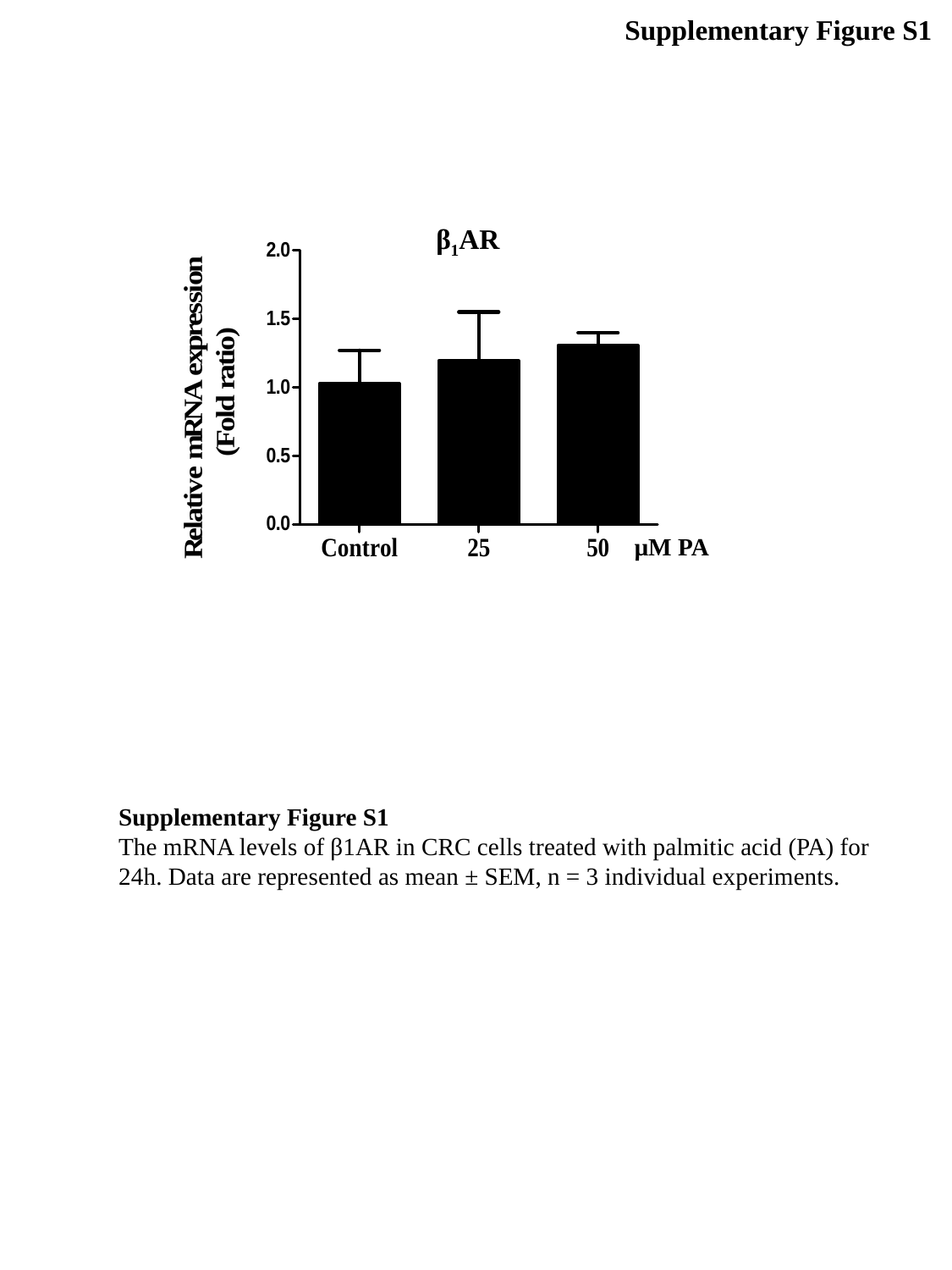

Supplementary Figure S1
β1AR
µM PA
Supplementary Figure S1
The mRNA levels of β1AR in CRC cells treated with palmitic acid (PA) for 24h. Data are represented as mean ± SEM, n = 3 individual experiments.

## Slide 2
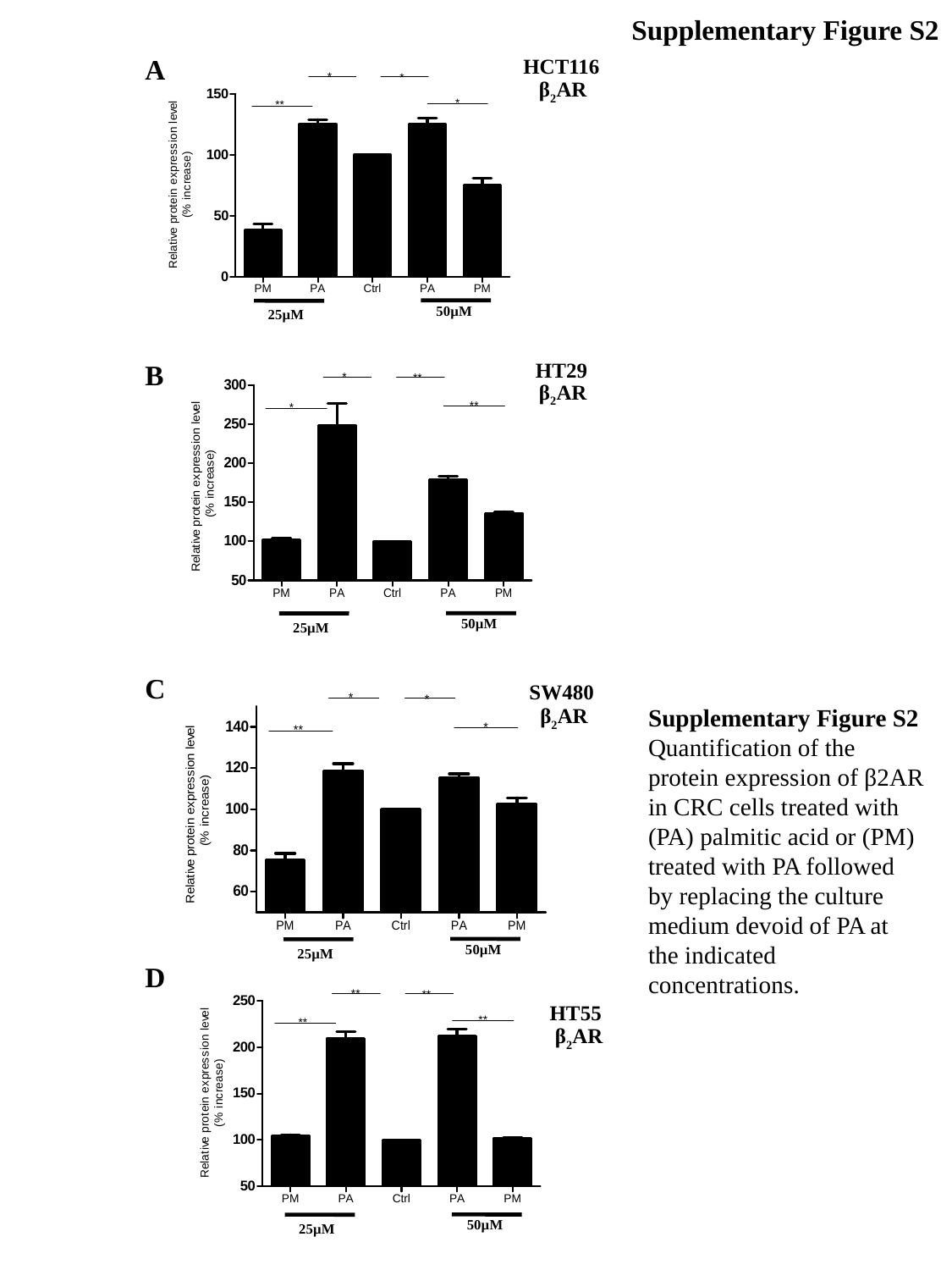

Supplementary Figure S2
A
HCT116
β2AR
50µM
25µM
HT29
B
β2AR
50µM
25µM
C
SW480
Supplementary Figure S2
Quantification of the protein expression of β2AR in CRC cells treated with (PA) palmitic acid or (PM) treated with PA followed by replacing the culture medium devoid of PA at the indicated concentrations.
β2AR
50µM
25µM
D
HT55
β2AR
50µM
25µM

## Slide 3
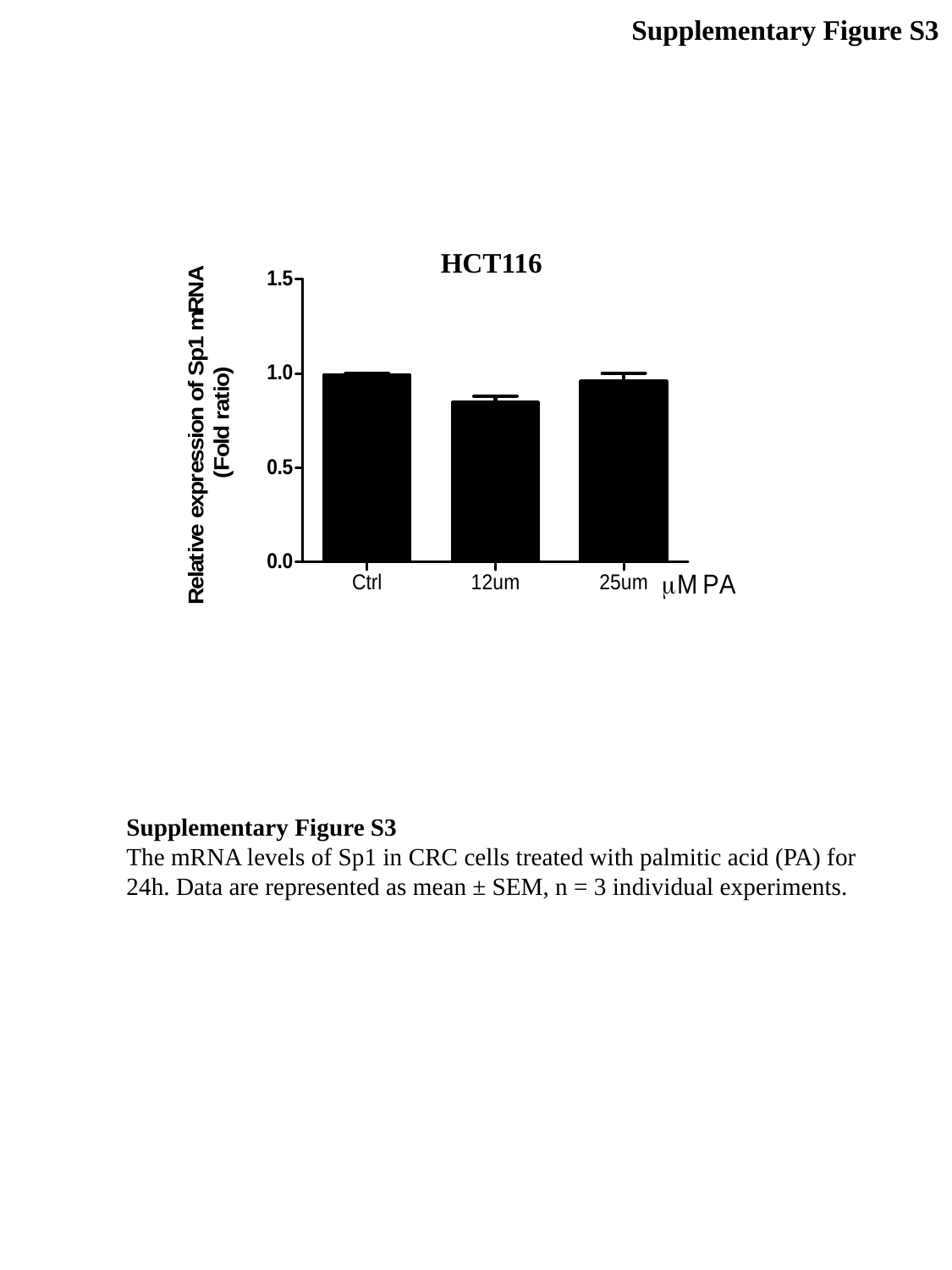

Supplementary Figure S3
HCT116
Supplementary Figure S3
The mRNA levels of Sp1 in CRC cells treated with palmitic acid (PA) for 24h. Data are represented as mean ± SEM, n = 3 individual experiments.

## Slide 4
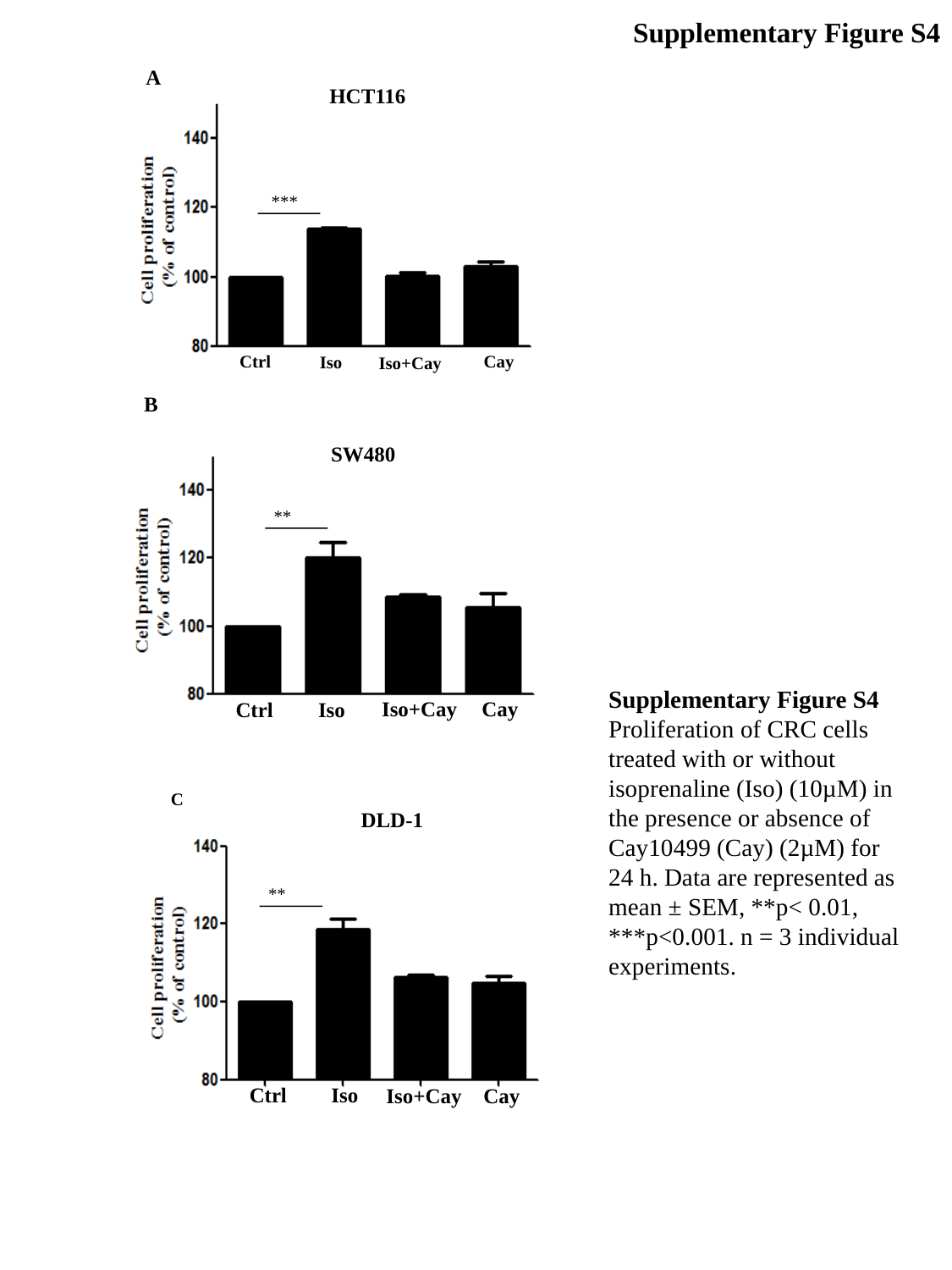

Supplementary Figure S4
A
Cay
Ctrl
Iso
Iso+Cay
HCT116
***
B
 Iso+Cay
Cay
Ctrl
Iso
**
SW480
Supplementary Figure S4
Proliferation of CRC cells treated with or without isoprenaline (Iso) (10µM) in the presence or absence of Cay10499 (Cay) (2µM) for 24 h. Data are represented as mean ± SEM, **p< 0.01, ***p<0.001. n = 3 individual experiments.
C
DLD-1
Ctrl
Iso
Iso+Cay
Cay
**

## Slide 5
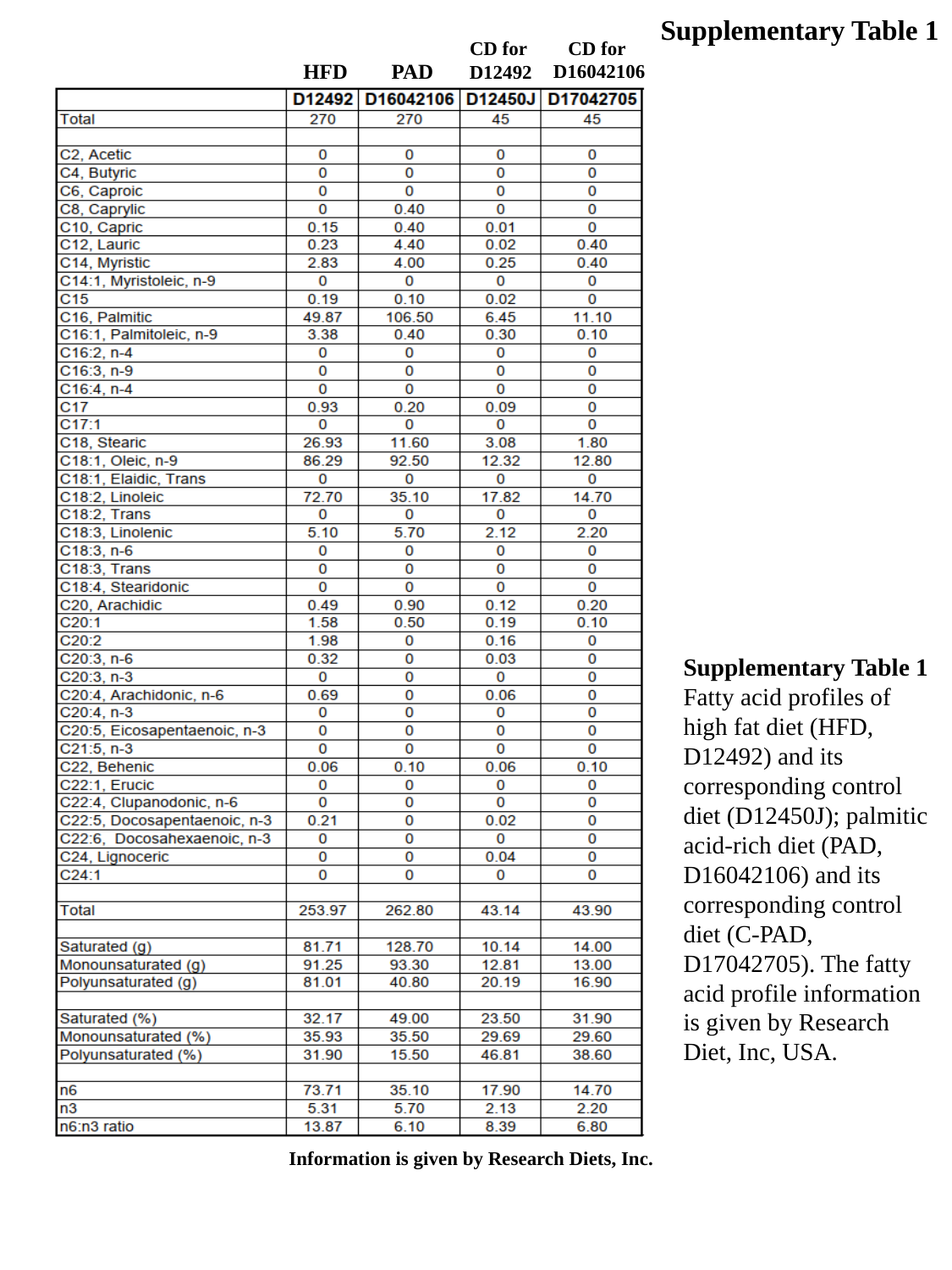

Supplementary Table 1
CD for
D16042106
CD for
D12492
HFD
PAD
Information is given by Research Diets, Inc.
Supplementary Table 1
Fatty acid profiles of high fat diet (HFD, D12492) and its corresponding control diet (D12450J); palmitic acid-rich diet (PAD, D16042106) and its corresponding control diet (C-PAD, D17042705). The fatty acid profile information is given by Research Diet, Inc, USA.
